# Human Retinal Progenitor Cells Derived Small Extracellular Vesicles Delay Retinal Degeneration: A Paradigm for Cell-free Therapy

**DOI:** 10.3389/fphar.2021.748956

**Published:** 2021-11-29

**Authors:** Min Chen, Chunge Ren, Bangqi Ren, Yajie Fang, Qiyou Li, Yuxiao Zeng, Yijian Li, Fang Chen, Baishijiao Bian, Yong Liu

**Affiliations:** ^1^ Southwest Hospital/Southwest Eye Hospital, Third Military Medical University (Army Medical University), Chongqing, China; ^2^ Key Lab of Visual Damage and Regeneration and Restoration of Chongqing, Chongqing, China; ^3^ Department of Medical Technology, Chongqing Medical and Pharmaceutical College, Chongqing, China; ^4^ Army 953 Hospital, Shigatse Branch of Xinqiao Hospital, Army Medical University, Shigatse, China

**Keywords:** human retinal progenitor cells, photoreceptor cells, microglia, apoptosis, inflammation, retinal degeneration, cell-free therapy, small extracellular vesicles (sEVs)

## Abstract

Retinal degeneration is a leading cause of irreversible vision impairment and blindness worldwide. Previous studies indicate that subretinal injection of human retinal progenitor cells (hRPCs) can delay the progression of retinal degeneration, preserve retinal function, and protect photoreceptor cells from death, albeit the mechanism is not well understood. In this study, small extracellular vesicles derived from hRPCs (hRPC-sEVs) were injected into the subretinal space of retinal dystrophic RCS rats. We find that hRPC-sEVs significantly preserve the function of retina and thickness of the outer nuclear layer (ONL), reduce the apoptosis of photoreceptors in the ONL, and suppress the inflammatory response in the retina of RCS rats. *In vitro*, we have shown that hRPC-sEV treatment could significantly reserve the low-glucose preconditioned apoptosis of photoreceptors and reduce the expression of pro-inflammatory cytokines in microglia*.* Pathway analysis predicted the target genes of hRPC-sEV microRNAs involved in inflammation related biological processes and significantly enriched in processes autophagy, signal release, regulation of neuron death, and cell cycle. Collectively, our study suggests that hRPC-sEVs might be a favorable agent to delay retinal degeneration and highlights as a new paradigm for cell-free therapy.

## Introduction

Retinal degeneration (RD), including retinitis pigmentosa (RP) and age-related macular degeneration (AMD), is characterized by the progressive degeneration of rod and cone photoreceptors, resulting in the gradual loss of vision and eventually blindness (Ferrari et al., 2011; Zhang et al., 2012). This group of retinal disorders shares the same pathological events including apoptosis, autophagy, and necrosis of photoreceptors (Boya et al., 2016). There are no therapies available to neither prevent the gradual loss of vision nor restore the damaged retina. Treatment of RP and AMD is largely an unmet need. Stem cell replacement therapy is a future direction that might provide a cure for RD. It has been reported that human retinal progenitor cells (hRPCs) transplanted into the subretinal space (SRS) of RD rats can maintain visual function (Wang et al., 2008). Our previous study demonstrates that hRPCs significantly improve the visual acuity of eight RD patients in a 6-month follow-up period after transplantation (Liu et al., 2017). hRPCs could promote the survival of photoreceptors in retinal neurodegenerative diseases that are largely due to the beneficial properties of hRPCs themselves including their ability to migrate and integrate into the damaged host retina and thus provide neuroprotection (Cuenca et al., 2014; Mollick et al., 2016). Although the underlying mechanism is unclear, recent studies have attributed the benefits to the paracrine effect of hRPCs, which mediate the therapeutic function of the mother cells before dividing to daughter cells (Ratajczak et al., 2006; Doorn et al., 2012; Luo et al., 2014; Ma’ayan Semo et al., 2016). Therefore, we speculate that small extracellular vesicles derived from hRPCs (hRPC-sEVs) may be the one that exerts neuroprotective effects in treating RD.

Extracellular vesicles (EVs) are the collective term for the vesicles secreted from eukaryotic cells (Xie et al., 2020). Exosomes/sEVs are phospholipid bilayer-enclosed nanoscale vesicles with size ranging from 30 to 150 nm and carry cell type–specific RNAs, microRNAs (miRNAs), biologically active proteins, and genetic material that play a major role in cell-cell communication (Kalluri and Lebleu, 2020; Ma et al., 2020; Priglinger et al., 2021). A study suggests that human neural progenitor cells protect retinal neurons from dystrophies by producing a multitude of neurotrophic factors (Mohlin et al., 2011). Our latest study demonstrates that the exosomes derived from grafted mouse neural progenitor cells could inhibit microglia activation and reduce inflammation in RD process and thus protect photoreceptors from apoptosis (Bian et al., 2020). The recent analysis reveals that human EVs could be internalized by mouse RPCs and transferred to the nucleus (Zhou et al., 2018). On the basis of the evidence mentioned above, we believe that SEVs derived from RPCs could be used to treat retinal degenerative diseases.

In our study, we inject hRPC-sEVs (20 μg/eye) once into the SRS of RCS rats to investigate the effect on the degenerating retina during the early 4 weeks after injection, especially on photoreceptors and microglia. We have shown that hRPC-sEVs could not only suppress microglia activation but also promote the survival of photoreceptors both *in vivo* and *in vitro*. Moreover, we have analyzed the miRNAs of hRPC-sEVs and predicted target genes that are related to inflammation, processes autophagy, signal release, regulation of neuron death, and cell cycle, which suggest that hRPC-sEVs contain abundant miRNAs to support photoreceptor survival and alleviate neuroinflammation. This study supports a paradigm that a cell-free sEVs-mediated therapy is effective in treating RD diseases.

## Materials and Methods

### Animals

The RCS rats used in this study were raised in the animal facility of the Southwest Eye Hospital, Third Military Medical University. The rats were reared according to a standard 12-h/12-h light/dark cycle. All animal experiments were approved by the Third Military Medical University Animal Care and Use Committee (no. AMUWEC20210132).

### Cell Isolation and Culture

Isolation and culture of hRPCs were performed as described in the study (Ma’ayan Semo et al., 2016). Primary hRPCs were isolated from human fetal neuroretina at 12–16 weeks of gestation. The retinas were provided from the embryonic tissue bank in the Department of Obstetrics at the Southwest Hospital, Army Medical University. All experiments involving human cells and tissues are conducted in accordance with the principles of the Declaration of Helsinki; the experiments of human tissues/cells were approved by the Ethics Committee of Southwest Hospital, Army Medical University (no. KY2019109). The neuroretina was enzymatically digested into a cell suspension with TrypLE Express (Gibco, 12604021). The RPCs were seeded in UltraCULTURE medium, supplemented with human epidermal growth factor (20 ng/ml), human basic fibroblast growth factor (10 ng/ml), and 1% penicillin-streptomycin. All cells were incubated in a 37°C 5% CO_2_ saturation humidity incubator. At passage 4, hRPCs were plated in T75 flasks for 6 h. The GFP virus was added to the complete medium and poured into hRPC-cultured well. After 24 h of incubation, the medium was replaced with fresh complete medium. GFP-labeled cells were detected with fluorescent microscopy.

For the glucose deprivation experiment, the 661W cells were cultured in six-well plates for 24 h with High-Glucose DMEM (HyClone) with 10% fetal bovine serum (FBS) (Gibco) and 1% penicillin-streptomycin (ScienCell) and were washed with phosphate-buffered saline (PBS) three times, and then, 661W cells were cultured with low-glucose serum-free medium treated with sEVs (60 µg/ml) or vehicle (PBS) as control for 48 h. Cells and supernatant are collected to apoptosis assays or lactate dehydrogenase (LDH) release assay (Emery et al., 2011).

BV2 and HMC3 were both cultured with High-Glucose DMEM (HyClone) with 10% FBS and 1% penicillin-streptomycin. Lipopolysaccharides (LPSs; Sigma-Aldrich, L4516) were supplemented to the medium (ultimate concentration, 1 μg/ml) for 4 h to stimulate microglia (Awada et al., 2014). Microglia were washed with PBS three times and then incubated with or without hRPC-sEVs (60 μg/ml) for 48 h. Afterward, the mRNA and culture media were collected for reverse transcription quantitative PCR (RT-qPCR) and ELISA assays.

### Flow Cytometry (FCM)

hRPCs were isolated as single-cell suspensions in TrypLE Express at a concentration of 1 × 10^6^/100 µl and were incubated with conjugated antibodies including PAX6 (BD Biosciences), Nestin (Invitrogen), SOX2 (BD Biosciences), and isotype antibody, as previously described (Schmitt et al., 2009). hRPCs were washed with Wash Buffer and resuspended with 300 µl of PBS and then transferred to a flow cytometry tube for FCM analysis. FlowJo software was used to analyze the data.

### Preparation and Characterization of Small EVs and PKH26 Labeling

Cell culture from proliferating hRPCs (passages 3–5) was collected and used for the isolation of sEVs. The culture supernatant was collected and centrifuged at 1,500 rpm for 10 min and at 2,500 rpm for 15 min at 4°C to remove cell debris. After centrifugation, the cell supernatant was filtered through 0.22-µm pores to eliminate large cellular debris and was ultracentrifuged at 110,000 g for 70 min, and the microspheres were washed with PBS and ultracentrifuged at 110,000 g for 70 min (Clotilde Théry, 2006; Thery et al., 2018). Finally, sEVs were resuspended in PBS for use. The sEVs were observed by transmission electron microscopy (TEM). To perform the nanoparticle tracking analysis (NTA) of sEVs, a NanoSight instrument was used according to the instructions of the manufacturer (NanoSight NS300), as previously described (Noble et al., 2020). Western blot (WB) was used to examine the EVs surface markers of CD9, CD63, and CD81. According to the recommendations of the manufacturer, the sEVs were labeled with PKH26 dye and tracked by fluorescence microscopy (Sigma-Aldrich). Total protein of EVs amount is measured by bicinchoninic acid assay (BCA) (Beyotime). The dose of sEV is 20 μg/eye *in vivo* and 60 μg/ml *in vitro*. To preserve the consistency of the experiment, fresh sEVs are used in our study.

### Subretinal Transplantation

To better observe the transplantation, we transfected hRPCs with GFP-lentiviral vectors and labeled hRPC-sEVs with PKH26. Then, 2 µl of concentrated hRPC-sEVs PBS solution (containing 20 μg of protein) were injected into the SRS per eye or 2 × 10^5^ hRPCs both in the temporal direction of the eye of postnatal week 3 RCS rats. The control group was injected with the same amount of PBS solution (n = 5 eye per group). All RCS rats, including vehicle group, received oral cyclosporine A (210 mg/L) (Sandoz, United Kingdom) dissolved in drinking water from day 7 before transplantation to day 14 after transplantation (Park et al., 2019).

### Scotopic Electroretinogram Recording

Electroretinogram (ERG) was recorded 1–4 weeks after the operation. RCS rats were dark-adapted for at least 16 h before the ERG test. RCS rats were abdominal general anesthesia. Then, the animals were anesthetized on the ocular surface and pupil dilation. The corneal electrodes were attached to the cornea as recording electrodes. The reference and ground electrode were placed under the skin of the cheek and tail. A strobe white stimulus was presented to the dark adaptation eyes; light stimuli were rendered at intensities of −2.5, −0.5, −0.02, 0.5, and 1 log(cd*s/m2); and the responses were recorded and stored using the RETI-scan system.

### Tissue Processing and Immunohistochemistry

To prepare the tissue, the eyecups of RCS rats were collected 1–4 weeks after operation, then fixed in 4% PFA for 2 h, and then stored overnight in 30% sucrose for dehydration. The tissue was frozen with optimal cutting temperature compound and stored at −80°C. Each group of tissues used the same horizontal angle to cross cut the optic disc; the frozen tissues were sectioned along the transplantation area of the eye through the optic nerve head (ONH), as previously described (Inoue et al., 2007). The tissue was sectioned with thickness of 16 μm, air-dried at room temperature, and stored at −20°C. For immunofluorescence assay, the sections were washed with PBS and blocked in PBS supplemented with 3% goat serum and 0.3% Triton X-100. Then, the primary antibodies were added to the sections overnight at 4°C. The next day, the sections were washed with PBS and incubated with secondary antibodies for 2 h at 37°C. Finally, the nuclei were counterstained with DAPI.

### Outer Nuclear Layer Thickness Analysis

The morphological changes of retina were observed after injection. The degree of retinal damage was assessed by measuring the thickness of ONL. The thickness quantification of the outer nuclear layer (ONL) was measured at six regions of retina: near the limbal on both sides recorded as 3 and −3; the graft region and the opposite region recorded as 2 and −2; the optic nerve area and the opposite region recorded as 2 and −2. The ONH was defined as the original location (recorded as 0). The thickness of ONL was measured by ImageJ.

### Lactate Dehydrogenase Release Assay

The experiment is to detect the release of LDH (Beyotime, C0016) in the supernatant of 661W cells. The experiment is divided into three groups: 661W cells cultured with High-Glucose DMEM with 10% FBS medium, 661W cells alone cultured with low-glucose medium, and 661W cells cultured with low-glucose medium within hRPC-sEVs (60 μg/ml) for 48 h. All supernatant was collected for detection and tested according to the protocol of the manufacturer.

### ELISA Assays

The enzyme-linked immunosorbent assays (ELISAs) were utilized to quantitate cytokines in cell culture medium. After LPS stimulation of microglia, the supernatant of microglia cell culture with or without hRPC-sEVs (60 μg/ml) treatment for 48 h was harvested for the assay. ELISAs, including levels of tumors necrosis factor–α (TNF-α) (DAKEWE, 1117202; BioLegend, 430,907), interleukin-1β (IL-1β) (DAKEWE, 1110122; DAKEWE, 1210122), and IL-6 (DAKEWE,1110602; DAKEWE, 1210602), were performed according to the instructions of the manufacturer. The results of ELISA were detected at 450 nm by using a microplate reader.

### Apoptosis Assays

We used AnnexinV/PI fluorescein isothiocyanate (FITC) staining to detect cell apoptosis. 661W cells treated with hRPC-sEVs were collected, incubated with Annexin V–FITC and PI to the cells according to the instructions of the manufacturer (BD Biosciences, 556547). Then, flow cytometry was used to detect the number of apoptotic cells, and the ratio of apoptotic cells to non-apoptotic cells was analyzed.

TUNEL (terminal deoxynucleotidyl transferase–mediated deoxyuridine triphosphate nick end labeling) (Beyotime, C1088) is a method for detecting apoptotic DNA fragmentation. The slides were incubated with DAPI (Solarbio, C0065) and TUNEL label solution using an *in situ* cell death detection kit. Slides were then observed under a fluorescence microscope and count positive cells.

### Total RNA Isolation and Real-Time Quantitative PCR

Total RNA was extracted from cells (661W, BV2, and HMC3 cells) or retinal issues using RNAiso Plus followed by chloroform, as per the instructions of the manufacturer. The cDNA was generated using a PrimeScript™ RT reagent kit with gDNA Eraser (Takara, RR047A). Real-time qPCR was performed on CFX96 system (Bio-Rad) by an SYBR Premix Ex TaqTM II kit (Takara, RR820A) to measure the expression of the primer sequences (Supplementary Table S1) of each gene.

### Western Blot

hRPC-sEV pellets were lysed in 1× RIPA buffer with protease inhibitor cocktail. Protein concentration was measured with a BCA Protein Assay Kit. The sample was separated using 10% sodium dodecyl sulfate–polyacrylamide gel electrophoresis gel, and the proteins were electroblotted to polyvinylidene difluoride membranes. The membranes were incubated with anti-rabbit CD9, CD63, and CD81 and anti-rabbit horseradish peroxidase (HRP) conjugated for secondary antibodies (SBI System Biosciences, EXOAB-KIT-1). Chemiluminescent detection was performed using a kit (Thermo Fisher Scientific, catalog no. 32106).

### Functional Enrichment Analysis

miRNA sequencing data are from a previous study (Bian et al., 2020). miRNA abundance analysis by small RNA sequencing. Gene Ontology (GO) enrichment analysis of the biological process (BP) and the Kyoto Encyclopedia of Genes and Genomes (KEGG) pathway analysis were obtained to predict the potential functions of hRPC-sEV miRNA target genes.

### Statistics

Each experiment is repeated in at least three biological samples (individually indicated in the figure legends). Data are presented as means ± SD. SPSS V22.0 software is used to perform statistical analysis on the data. The statistical differences are measured with unpaired two-tailed Student’s t-test for comparison between two groups or analysis of variance among multiple groups by Dunnett’s T3 multiple comparison tests or Turkey’s t-test. *p*-value < 0.05 was considered statistically significant.

## Result

### Transplantation of hRPC-sEVs Preserves Visual Function in RCS Rats

Retina progenitor cells obtained from the neural retina of human fetal eyes, which are identified as positive for expression of PAX6, SOX2, and Nestin and weakly expression of GFAP (Supplementary Figure S1A,B), as previously described (Schmitt et al., 2009). The TEM examination shows that the hRPC-sEVs are cup-shaped vesicles, with the diameter about 130 nm (Supplementary Figure S2A). WB analysis demonstrates that the hRPC-sEVs positively express characteristic markers CD9, CD63, and CD81 (Supplementary Figure S2B). NTA shows that these nanoparticles have a particle size distribution between 20 and 150 nm (Supplementary Figure S2C).

**FIGURE 1:**
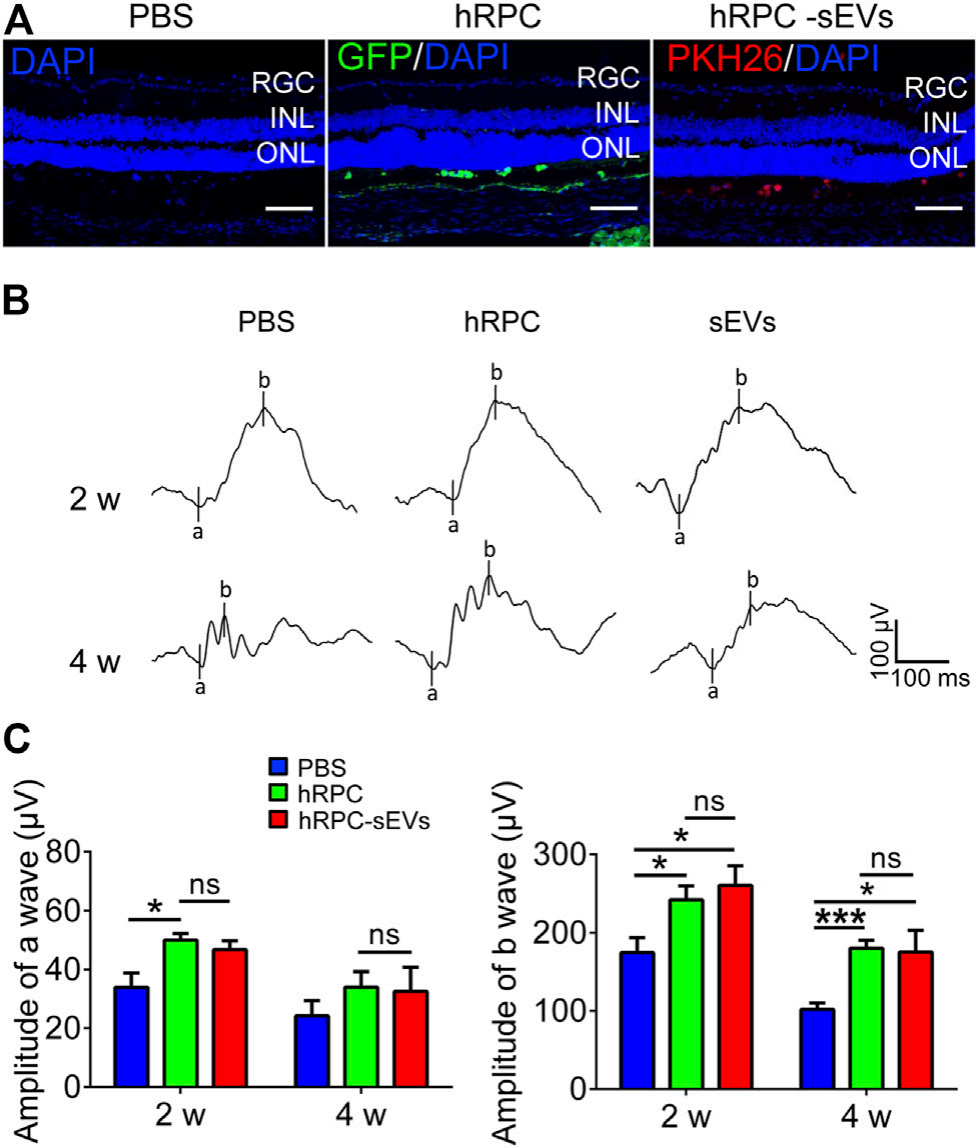
Effects of hRPCs and hRPC-sEV treatment on visual function in RCS rats. **(A)** Confocal images showing RCS retina injected with PBS (L), GFP-labeled (green) hRPC (M), or PKH26-labeled (red) hRPC-sEVs (R) in subretinal space. Scale bars, 50 μm. **(B)** Representative scotopic fERG waveforms elicited at 1 log(cd*s/m^2^) light intensity in PBS (untreated groups), hRPCs, and hRPC-Exos groups at 2 and 4weeks after injection (n = 5 eyes). **(C)** Statistical analysis of scotopic fERG a-wave amplitudes **(left panel)** and b-wave amplitudes **(right panel)** elicited at 1 log(cd*s/m^2^) light intensity in PBS (untreated groups), hRPCs, and hRPC-sEVs groups at 2 and 4 weeks after injection (n = 5). Data are shown as means ± SEM. *p < 0.05, **p < 0.01, and ***p < 0.001.

To demonstrate the therapeutic effects of hRPCs and their sEVs, we then give one injection of hRPCs and hRPC-sEVs, with PBS as the negative control to the RCS rats, and observe until 2 weeks after injection (Figure1A). Scotopic fERG is performed to determine vision outcome of RCS rats. The visual function assessed by ERG indicate a progressive deterioration of the a- and b-wave amplitudes 4 weeks after transplantation in all three groups caused by RD. We find that, compared with the PBS group, both hRPC group and hRPC-sEV group significantly preserve the amplitudes of b-waves at 2 weeks after injection, and this effect has maintained to 4 weeks after injection (Figure 1B). Although both hRPC-sEVs and hRPCs transplanted into RCS rats could significantly increase the amplitude of b-waves at 2 and 4 weeks after injection, the effect of two groups shows no significant difference (Figure 1C). These results demonstrate that, in the early stage of RD, the direct administration of hRPC-sEVs could preserve visual function, and the effect of RCS rats is as good as hRPCs.

### hRPC-sEVs Protect Photoreceptors From Apoptosis *in vivo*

**FIGURE 2:**
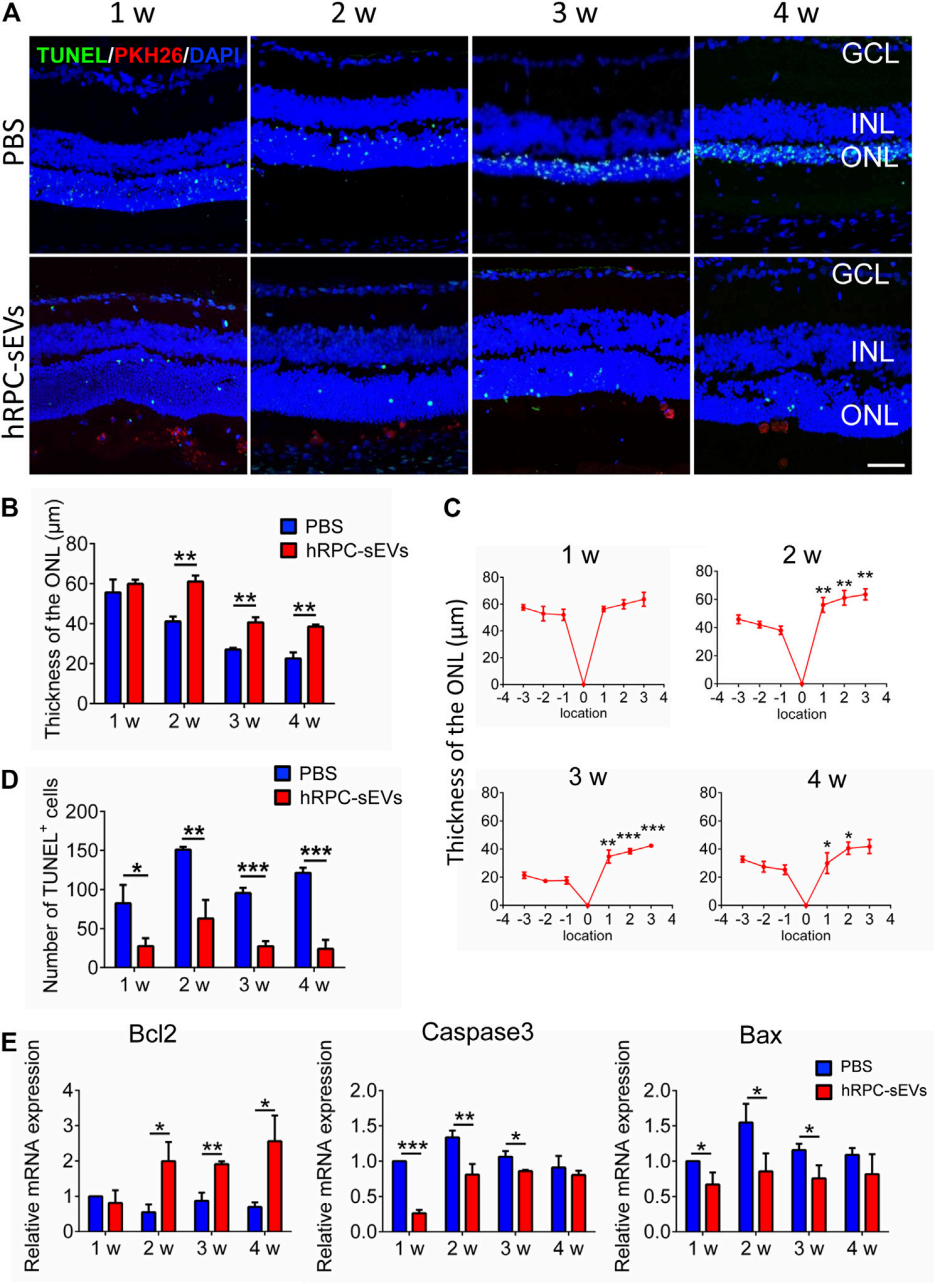
hRPC-sEVs protect photoreceptors from apoptosis in RCS rats. **(A)** Apoptosis detection of TUNEL and DAPI staining in hRPC-sEV– and PBS-treated RCS retinas at 1 to 4 weeks after injection. hRPC-sEVs are prelabeled with PKH26 (red). Scale bar, 50 μm. **(B)** Thickness of the ONL at the injected sites in hRPC-sEV and PBS groups at each time point. The thickness of ONL around the injected area is significantly preserved in the hRPC-sEV group 2 weeks after injection compared to the PBS group, and this effect has maintained to 4 weeks after injection (n = 3). **(C)** The thickness of ONL is compared in hRPC-sEVs at different injected bitamporal locations, distance from optic nerve head (ONH), from 1, 2, 3, and 4 weeks after transplantation (n = 3). The thickness of the ONL in the transplanted area (temporal field) is significantly preserved compared to that of the distal area (nasal field). **(D)** The numbers of TUNEL-positive cells in the retina of RCS rats are analyzed in hRPC-sEV and PBS groups at each time point (n = 3). hRPC-sEV treatment significantly reduces the number of TUNEL-positive cells around the injected area of eyes in RCS rats. **(E)** Real-time qPCR analysis showing relative mRNA expression of apoptotic factors Bcl2, Caspase3, and Bax in the retinas of the hRPC-sEV and PBS groups. hRPC-sEVs significantly reduce the apoptotic factors Bax and Caspase3 and markedly increase the antiapoptotic factor Bcl2 in the retina compared to the PBS group (n = 3). Data are shown as means ± SD. *p < 0.05, **p < 0.01, and ***p < 0.001.

It has been reported that RCS rats suffer from photoreceptor loss (Lew et al., 2020). Therefore, the ONL thickness is analyzed to investigate the protective function of the grafted hRPC-sEVs (Figure 2A). We find that, compared with the PBS group, the ONL thickness around the injected area is significantly retained in the hRPC-sEV group 2 weeks after injection, and this effect has maintained to 4 weeks after injection (Figure 2B). The thickness of ONL has been measured, and we find that the thickness of the ONL in the transplanted area (temporal field) is significantly preserved compared to that of the distal area (nasal field) (Figure 2C) at 4 weeks after injection. It is proved that hRPC-sEVs have a protective effect on the ONL of retina, and this effect is limited to the local areas.

Moreover, TUNEL staining reveals that hRPC-sEV treatment significantly reduce the number of TUNEL-positive cells around the injected area of eyes at 4 weeks after injection in RCS rats (Figure 2D). Furthermore, RT-qPCR shows that hRPC-sEVs significantly reduce the apoptotic factors Bax and Caspase3 and markedly increase the antiapoptotic factor Bcl2 in the retina compared to the PBS group (Figure 2E). These observations demonstrate that the grafted hRPC-sEVs protect photoreceptors from apoptosis and delay the progressivity of RD.

### hRPC-sEVs Inhibit Low-Glucose–Induced Photoreceptor Apoptosis *in vitro*

**FIGURE 3:**
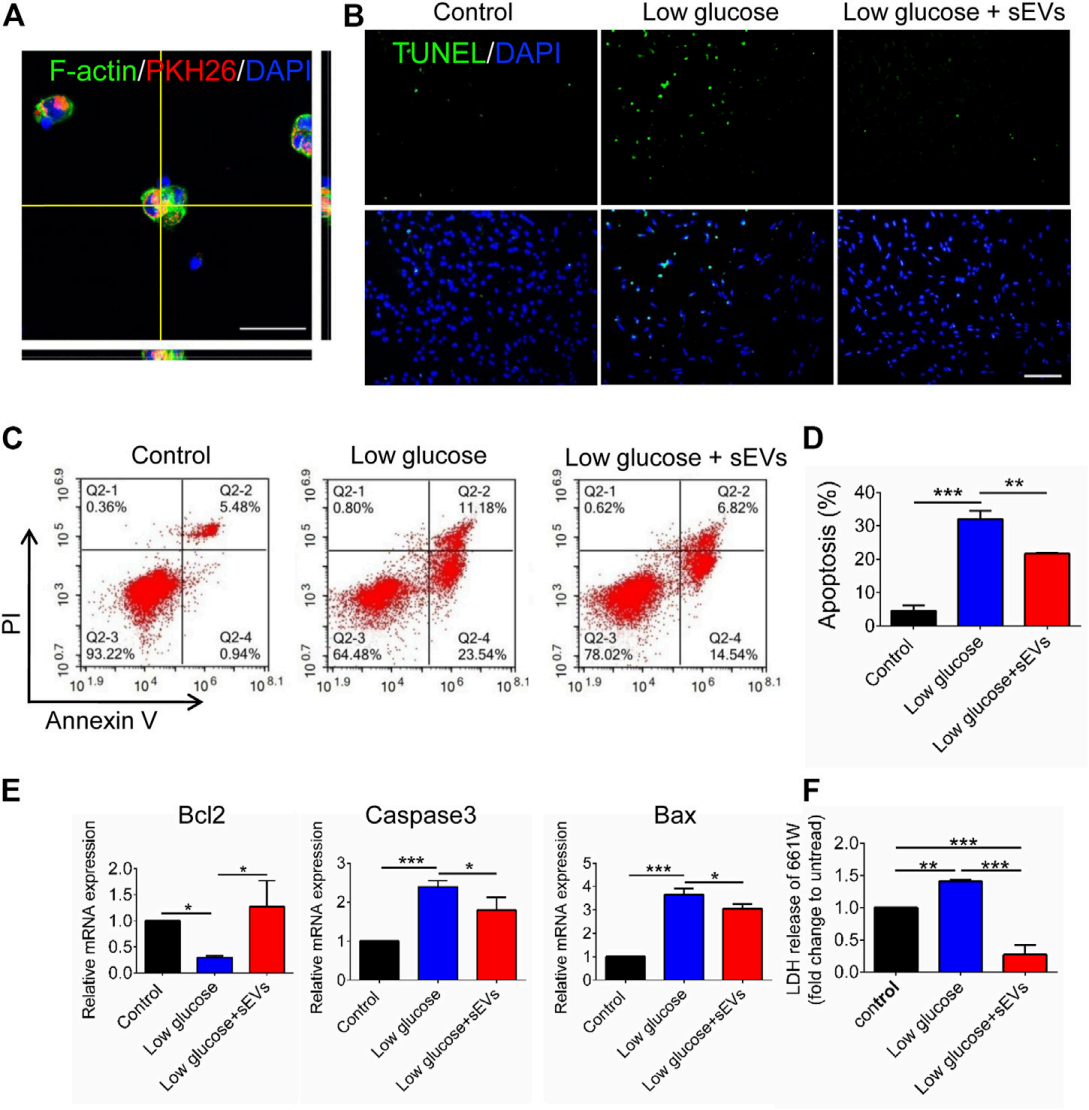
hRPC-sEVs protect 661W cells from apoptosis in low-glucose preconditioned culture. **(A)** Representative confocal images showing the hRPC-sEVs (PKH26-labeled, red) are localized with Factin-labeled mouse 661W cells (green). **(B)** Confocal images of TUNEL (green, **top panel**) and DAPI (blue, **bottom panel**) staining in low-glucose cultured 661W cells. hRPC-sEV–treated groups show low TUNEL-positive labeling compared with the PBS groups (n = 3). Scale bar, 50 μm. **(C)** Flow cytometry representative images showing Annexin V/PI staining in control (normal glucose), low glucose without treatment, and low glucose with treatment of hRPC-sEVs (n = 3 per group). The double-positive cells are end-stage necrotic cells. A lower percentage of Q2-2 cells (6.82%) in hRPC-sEV–treated group compared to non-treated group (11.18%), suggesting that hRPC-sEVs are protecting 661W cells from death. **(D)** Statistical analysis of the apoptosis assays from flow cytometry testing (n =3). **(E)** Real-time qPCR analysis showing relative mRNA expression of apoptotic factors Bax, Caspase3, and Bcl2 in the 661W cells. hRPC-sEV–treated 661W cells significantly reverse the high levels of Bax and caspase3 and the low level of Bcl2 (n = 3). **(F)** LDH release of 661W cells (n = 3). hRPC-sEV treatment dramatically reduces the LDH release in 661W cells compared to the untreated group (n = 3 per group). Data are shown as means ± SD. *p < 0.05, **p < 0.01, and ***p < 0.001.

To investigate the protective on degenerating photoreceptor cells, we establish a low-glucose–induced apoptosis model of 661W cells. The 661W cells, a mouse photoreceptor cell line, as previously described (Emery et al., 2011). After incubation with hRPC-Exos for 6 h, the PKH26-labeled hRPC-sEVs are shown toco-locate with 661W cells, which suggest that the photoreceptors are interacting directly with hRPC-sEVs (Figure3A). TUNEL assay shows that an exposure to low glucose significantly increases apoptosis compared to the untreated group, whereas the administration of hRPC-sEVs significantly attenuates the apoptosis in low-glucose–treated 661W cells (Figure 3B). Flow cytometry also shows a higher cell death percentage at 24 h low-glucose preconditioning compared to the control group, whereas treatment with hRPC-sEVs significantly mitigates low-glucose–induced cell death in photoreceptors compared to the untreated group. This result is consistent with TUNEL assay (Figure 3C,D). RT-qPCR shows that Bax and Caspase3 are upregulated and Bcl-2 is downregulated compared to the control in 661W cells after 24 h of low-glucose preconditioning, whereas hRPC-sEV–treated 661W cells significantly reverse the high levels of Bax and Caspase3 and the low level of Bcl2 (Figure 3E). 661W cells preconditioned with low glucose for 24 h lead to dramatic release of LDH to the medium. hRPC-sEV treatment dramatically reduces the LDH release in 661W cells compared to the untreated group (Figure 3F). The results indicate that hRPC-sEVs could be taken up and internalized by 661W cells and protect photoreceptor from low-glucose–induced apoptosis *in vitro*.

### hRPC-sEVs Inhibit Microglia Activation and Mitigate Ocular Inflammation

**FIGURE 4:**
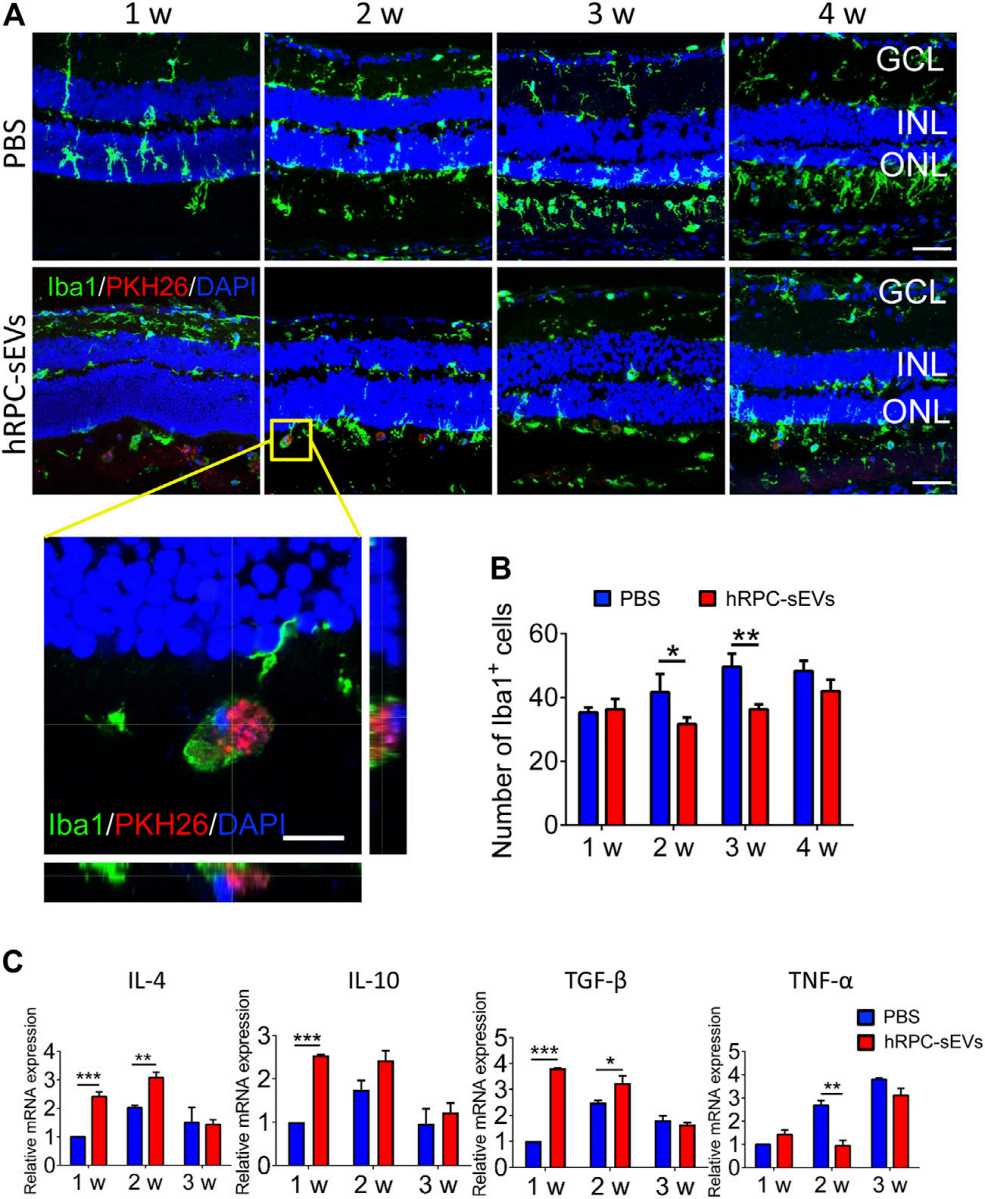
hRPC-sEVs suppress the activation of microglia and regulate cytokines in the retina of RCS rats. **(A)** Iba1 (green) staining of PBS-treated **(top panel)** and hRPC-sEV–treated **(bottom panel)** RCS retinas at different time after transplantation. hRPC-sEVs are labeled by PKH26 (red). Enlarged orthogonal view of hRPC-sEVs at the injected site shows that the EVs are co-located with microglia in subretinal space (SRS). **(B)** The number of Iba1-positive cells in the injected area of the retina is analyzed and compared in hRPC-sEV– and PBS-treated groups at different time points (n = 3 per group). **(C)** Relative mRNA levels of cytokines IL-4, IL-10, TGF-β, and TNF-α in the retinas (n = 3 per group). Data are shown as means ± SD. *p < 0.05, **p < 0.01, and ***p < 0.001.

To investigate the effect of sEVs on microglia in RD, microglia is detected with Iba-1 immunofluorescence staining. Microglia is observed to migrate from the inner nuclear layer to SRS and co-locate with hRPC-sEVs (Figure 4A). Compared to the PBS group, the number of migrated microglia in hRPC-sEV–treated group is markedly decreased at 4 weeks after injection, in both ONL and SRS. Retinal treatment with small EVs has markedly reduced the number of microglia in the ONL of the retina (Figure 4B). Microglial cells migrate from the inner to the outer retina in RD progress, which symbolizes their activation in response (Cuenca et al., 2014). The proliferation of microglia is often corresponding with excessive production of inflammatory cytokines. After the small EV treatment, we find that gene expressions of IL-4, IL-10, and transforming growth factor–β (TGF-β) were significantly increased, and TNF-α is reduced in the retina compared to PBS-treated RCS rat (Figure 4C). The results suggest that hRPC-sEVs could inhibit microglia activation and mitigate the inflammatory response in RCS rats.

### hRPC-sEVs Reduce the Expression of Pro-inflammatory Cytokines in Microglia *in vitro*

**FIGURE 5:**
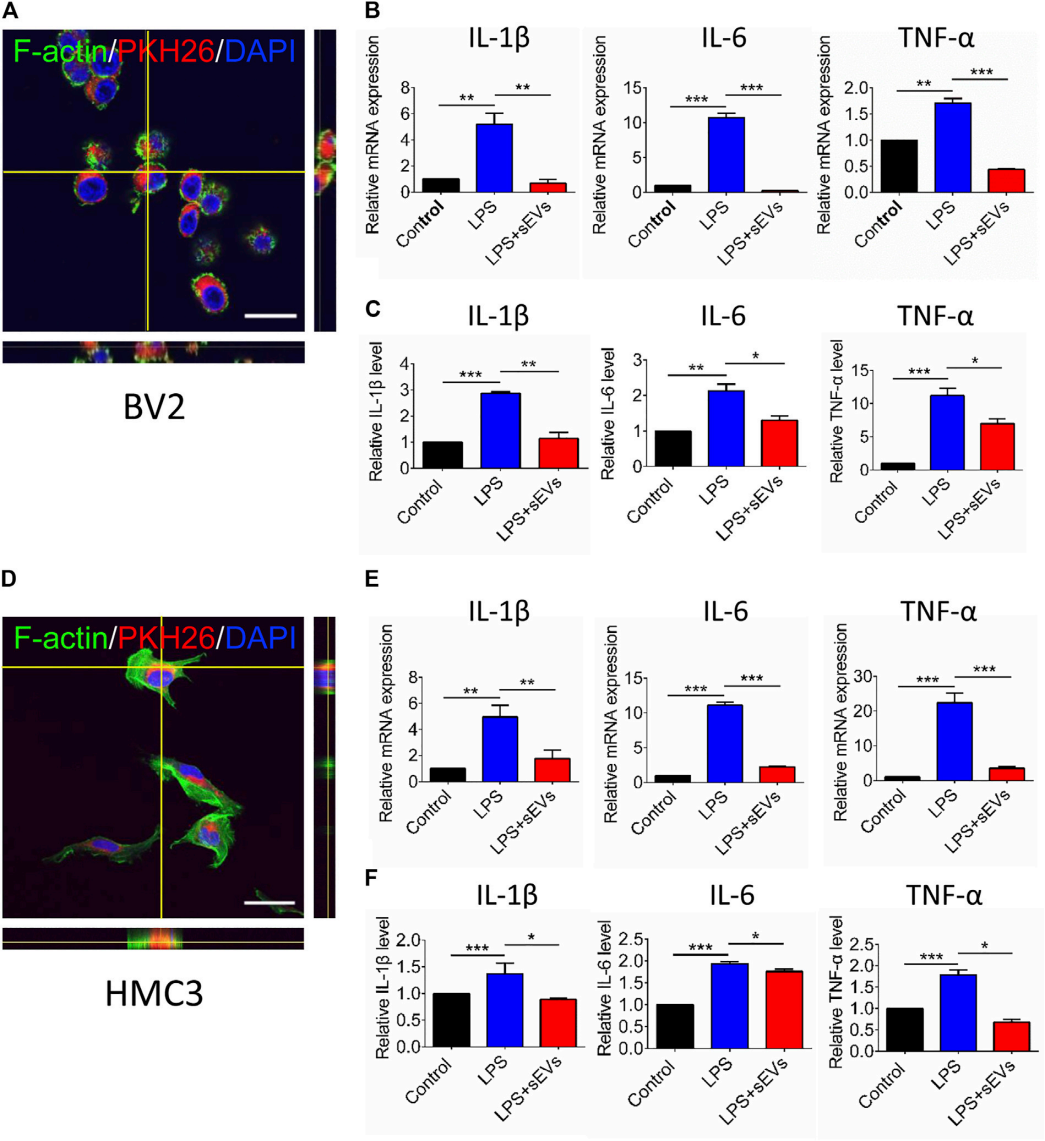
Microglia phagocytosis assay of hRPC-sEVs and cytokines changes after LPS stimulation of microglia. Representative confocal images showing the hRPC-sEVs (red) in the medium are located with Factin-labeled mouse **(A)** or human **(D)** microglia cells (green). Enlarged orthogonal view shows that the cytoplasm of microglia contains a large number of hRPC-sEV particles after LPS stimulation. Scale bar, 50 μm. Real-time qPCR analysis showing relative mRNA expression of cytokines IL-1β, IL-6, and TNF-α in cultured mouse microglia **(B)** and human microglia **(E)**. Analysis of cytokine concentrations such as TNF-α, IL-6, and IL-1β in the supernatant of mouse microglia **(C)** and human microglia. **(F)** Culture detected by ELISA. Data are shown as means ± SD. *p < 0.05, **p < 0.01, and ***p < 0.001.

Because the production and release of pro-inflammatory cytokines are essential in microglia-mediated inflammation. we next determine the gene and protein expression of pro-inflammatory mediators produced by activated microglia *in vitro*. First, PKH26-labeled small EVs are co-cultured with BV2 and HMC3 cells for 6 h. The labeled sEVs co-localized with BV2 cells are mainly located in the perinuclear region within the BV2 cell margins (Figure 5A). We find that LPS stimulation significantly increases the production of TNF-α, IL-1β, and IL-6. However, these pro-inflammatory effects are significantly attenuated by co-culture with hRPC-sEVs, in both gene and protein levels. We concluded that hRPC-sEVs inhibit neuroinflammation by stabilizing microglia not to release cytokines (Figures 5B,C). The labeled sEVs also co-localized with HMC3 cells (Figure 5D). A consistent trend was found in HMC3 group that both gene and protein levels of pro-inflammatory cytokines significantly decreased in hRPC-sEV–treated HMC3 cells (Figures 5E,F). These results show that, both in mouse and human cell lines, hRPC-sEVs inhibit neuroinflammation by inhibiting microglia activation and suppress secreting cytokines.

### GO and KEGG Analysis of hRPC-Exos/sEVs miRNAs

**FIGURE 6:**
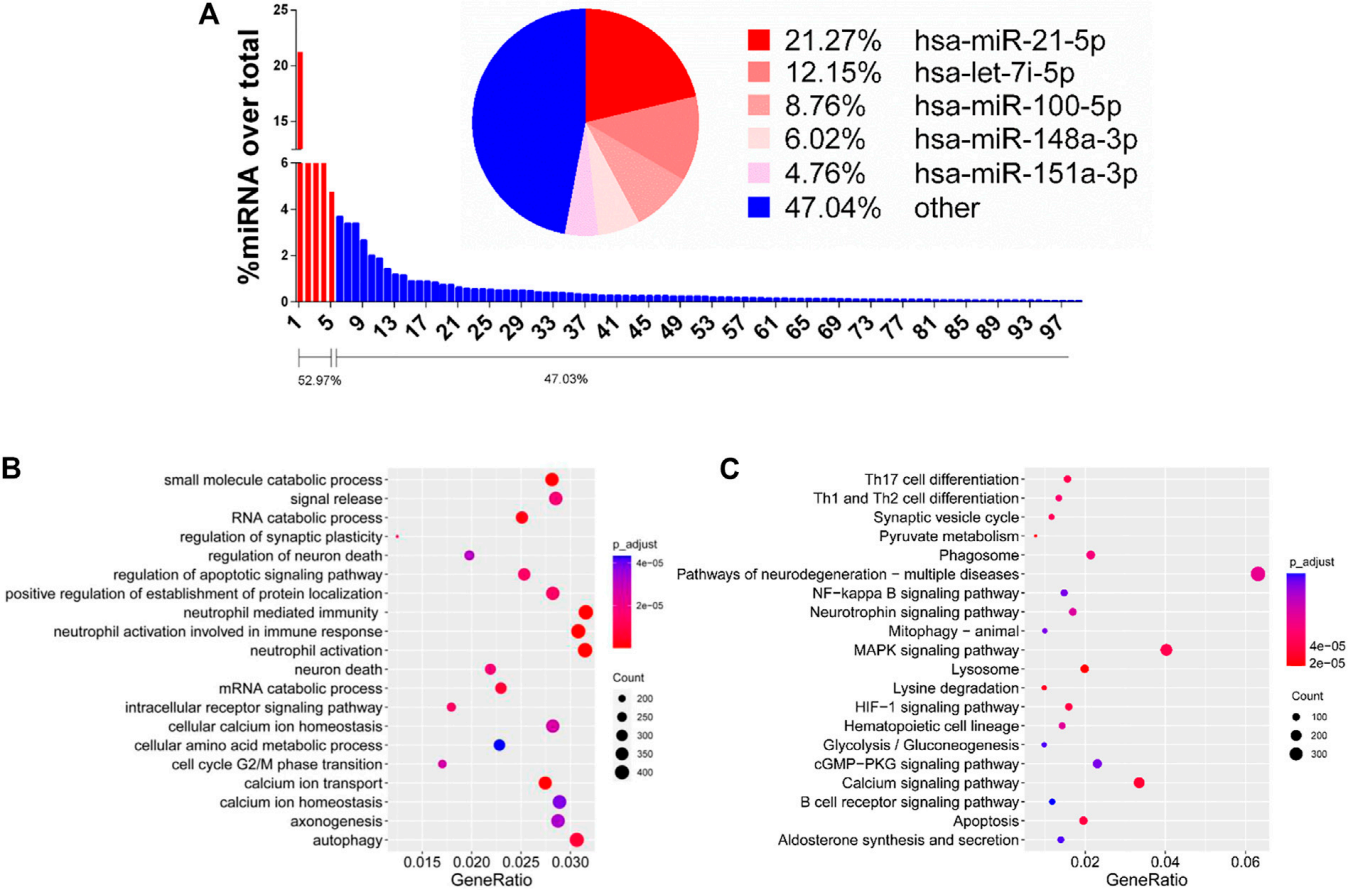
Functional analysis of EVs/Exo-miRNAs. **(A)** Bar diagram showing miRNAs expressed in theen riched hRPC-sEVs. Left to right is showing high to low percentages. The pie chart shows the top five miRNAs in hRPC-sEVs. Selected enriched GO biological processes **(B)** and KEGG pathways **(C)** for the top expressed miRNAs in hRPC-sEVs. The coloring of the q-values indicates the significance of the rich factor. The circle indicates the target genes that are involved, and the size is proportional to the gene numbers. The *X*-axis indicates the rich factor, which refers to the ratio of the number of genes located in the differentially expressed gene to the total number of the annotated genes located in the pathway. The *Y*-axis indicates the names of enriched pathways.

Small EVs contain cell-specific proteins or mRNA/miRNA that can interfere host tissue homeostasis and function (Geis-Asteggiante et al., 2018). Through analysis of small RNA sequencing, we are able to show the abundant expressed miRNAs in enriched hRPC-sEVs (Supplementary Table S2). The pie chart shows the top five miRNAs expressed in the hRPC-sEVs. It is found that the miRNAs mainly contained in hRPC-derived small EVs are miR-21-5p, let-7i-5p, miR-100-5p, miR-148a-5p, and miR-151a-3p, which take proportion of over 50% (Figure 6A). Subsequently, GO and KEGG enrichment pathway analyses are used to predict the target genes of top 20 expressed miRNAs in hRPC-sEVs (listed in Supplementary Table S2). The functions of the miRNAs (highly expressed in hRPC-sEVs) are predicted to be involved several signaling pathways (listed in Table 1) (Ouyang et al., 2019; Fu et al., 2020; Wei et al., 2020; Wen et al., 2020; Zhang H. et al., 2020; Zhang Y. et al., 2020; Li et al., 2021). Some of the enriched GO BPs are related to inflammation, such as neutrophil mediated immunity, neutrophil activation in immune response, and neutrophil activation (Nabavi et al., 2017; Liang et al., 2020). Moreover, processes autophagy, signal release, regulation of neuron death, and cell cycle are also significantly enriched (Figure 6B). KEGG analysis of the top 20 enriched pathways suggests that they are related to miRNAs targets in MAPK signaling pathway and pathway of neurodegeneration-multiple disease (Figure 6C).

**TABLE 1:** List of direct targets of selected miRNAs highly expressed in hRPC-sEVs and their functional role.

miRNA	Functions	Study
Hsa-miR-21-5p	inhibit cardiac microvascular endothelial cell apoptosis	Liao et al. (2021)
	regulated the proliferation and apoptosis	Zhang et al. (2020b)
	anti-inflammatory effect	Ouyang et al. (2019)
Hsa-let-7i-5p	attenuate hypoxia-induced apoptosis	Zhang et al. (2020a)
Hsa-miR-100-5p	protected cell from pyroptosis and injury	Liang et al. (2020)
		Nabavi et al. (2017)
Hsa-miR-26a-5p	reduce the apoptosis of myocardial cells and the expression of inflammatory factors	Li et al. (2021)
		Wen et al. (2020)
Hsa-miR-151a-3p	inhibited cell viability and promoted lactate dehydrogenase release	Fuet al. (2020)

## Discussion

In this study, we demonstrate that the hRPC-sEVs significantly preserve the function of retina and thickness of the ONL, reduce the apoptosis of photoreceptors, inhibit microglia activation and mitigate ocular inflammation, and delay the degeneration of the retina in RCS rats*. In vitro*, we have shown that hRPC-Exos treatment could significantly reserve the low-glucose preconditioned apoptosis of photoreceptors and reduce the expression of proinflammatory cytokines in microglia.

Microglia cells, as the reaction cells of retinal cell damage, participate in injury repair and inflammatory status (Xie et al., 2019). A persistent chronic pro-inflammatory environment is an important common feature of retinal degenerative diseases and neurological diseases that affect vision (Chen and Xu, 2015; Zhao et al., 2015; Ramirez et al., 2017). Therefore, regulating microglial reactivity has become a promising therapeutic approach (Simons and Raposo, 2009; Fruhbeis et al., 2013). In recent studies, exosomes are one of therapeutic approaches to show huge potential for neurological diseases (Alvarez-Erviti et al., 2011; Doeppner et al., 2015; Tatullo et al., 2020). Furthermore, neuronal EV as an endogenous protective factor inhibits microglial phagocytosis by targeting platelet-activating factor receptor, thereby reducing ischemia-induced neuronal death (Yang et al., 2021). Our recent study has found that RPCs from human embryonic stem cell–derived retinal organoids inhibit the activation of microglial, gliosis, and the production of inflammatory mediators (Zou et al., 2019). In addition, exosomes/sEVs derived from neural stem cells could significantly inhibit the activation of microglia and protect photoreceptors from apoptosis (Bian et al., 2020). Therefore, we are interested in the effect of hRPC-sEVs on microglia in RCS rats. In our study, we have observed that small EVs can be internalized by microglia in the retina. Moreover, we have compared the number of microglia and the expression of inflammatory factors in the retina with or without sEVs treatment. The results confirm that hRPC-sEVs can protect the degenerating photoreceptors from death in the RD model by suppressing microglia activation. Our work further supports the idea that exosomes inhibit neuroinflammation by inhibiting the activation of microglia in neurodegenerative diseases (Bian et al., 2020).

We have observed that hRPC-sEVs exerts a good anti-apoptosis effect on damaged photoreceptors. It is reported that the transplanted stem cells protect retina from damage via multiple mechanisms, and the exchange of therapeutic cargoes to host cells via extracellular vehicles is one of the most important mechanism. The miRNA of small EVs is one of the therapeutic mechanisms of acellular therapy in the retina (Wang et al., 2020; Peruzzotti-Jametti et al., 2021). Exosomes can be used to deliver interfering miRNA, siRNA, or drug active substances (Alvarez-Erviti et al., 2011; Zhuang et al., 2011; Jiang et al., 2020). Therefore, it is speculated that small EVs can reduce cell apoptosis by transferring their functional substances miRNAs. Our result shows that the main components (>50%) of miRNAs in hRPC-Exos are miR-21-5p, let-7i-5p, miR-100-5p, miR-148a-5p, and miR-151a-3p. Previous studies have reported that miR-21-5p of cardiac telocyte–derived exosomes can inhibit apoptosis in cardiac microvascular endothelial cells (Liao et al., 2021). Enriched miR-100-5p in human umbilical cord mesenchymal stem cell–derived exosomes protects against H/R-induced cardiomyocyte pyroptosis and injury through suppressing FOXO3 expression (Liang et al., 2020). Overexpressing let-7i-5p could attenuate hypoxia-induced apoptosis and mitochondrial energy metabolism dysfunction in AC16 cells (Zhang et al., 2020b). MiR-26a-5p could target and regulate ADAM17 and reduce the apoptosis of myocardial cells and the expression of inflammatory factors in acute myocardial infarction (Wen et al., 2020). These miRNAs are known to be associated with cell proliferation, anti-inflammatory effect, and apoptosis and play a significant role in regulation of inflammation and cell apoptosis. These findings are consistent with prediction of the target genes, which may be the mechanism to inhibit microglial activation and alleviate ocular inflammation. Further studies are therefore essential to confirm the function of these miRNAs and their targets in neurodegenerative diseases.

Considerable benefit of EVs is that it does not contain cell nucleus as a treatment agent and is storable (Kusuma et al., 2018; Mead and Tomarev, 2020). EV therapy can keep treatment effect of the stem cells using non-living cell products (Luga et al., 2012) and is free of concerning viable cell risk such as tumor genesis or complications associated with the stem cell transplant (Kuriyan et al., 2017; Huang et al., 2019). As previously described, EVs showed minimal toxicity and immunogenicity about systemic administration or repeated dosing in mice (Zhu et al., 2017; Saleh et al., 2019; Rodrigues et al., 2021). The advantage of EVs is that the lipid bilayer composition protects encapsulated cargo from degradation and has been widely studied as drug delivery vectors exploiting natural properties (Ge et al., 2014; Li et al., 2015; Kooijmans et al., 2016; Sunkara et al., 2016). It has been reported that small EVs/exosomes are storable at −80°C for more than 6 months while maintaining functions (Clotilde Théry, 2006; Aryani and Denecke, 2016; Mead et al., 2018). For these reasons, small EV approach is valuable to be translated into clinical application.

Although hRPC-sEVs have a good application prospect in neurodegenerative diseases, exosomes as cell derivatives have some limitations waiting to be solved (Kooijmans et al., 2016; Shao et al., 2020). Quantification of EVs; timing, dose, and duration of treatment; underlying mechanisms, etc., remain to be optimized and elucidated (Buschmann et al., 2021). hRPC-sEVs are locally injected in the SRS of RCS rats in this study, and the therapeutic effects remained 4 weeks after injection. However, the pharmacokinetics of the grafted sEVs in SRS remains unclear. Lai et al. and Sung et al. have reported the system to track the EV biogenesis, uptake, and intracellular transport via a live cell reporter system (Lai et al., 2015; Sung et al., 2020). EVs are also reported to be administered intravenously and peritoneally, and with the latest imaging technologies, we can review the biodistribution of grafted EVs (Takahashi et al., 2013; Gupta et al., 2020). Thus, improving the route of administration, studying pharmacokinetics, developing slow-release reagent for hRPC-sEVs application, and so on, may be beneficial for application in diseases in RD.

In summary, we demonstrate that the hRPC-sEVs is a favorable cell-free therapy to treat retinal degenerative diseases in the short term, as it could significantly preserve the function of retina and thickness of the ONL, reduce the apoptosis of photoreceptors, and inhibit microglia activation and mitigate ocular inflammation, although it is a short-term treatment. Microglia internalization of therapeutic miRNAs cargoes from small EVs is one of the mechanisms that exert neuroprotective effect. Our study highlights the potential of hRPC-sEVs as cell derivatives therapeutic for neuroprotective and regenerative therapy in retinal degenerative disease and provides a new paradigm for cell-free therapy.

## Data Availability

The original contributions presented in the study are included in the article/Supplementary Material, further inquiries can be directed to the corresponding authors.
