# Supplementary material for: Human Retinal Progenitor Cells Derived Small Extracellular Vesicles Delay Retinal Degeneration: A Paradigm for Cell-free Therapy
Source: Front Pharmacol. 2021 Nov 29;12:748956. doi: 10.3389/fphar.2021.748956 (PMC8667779; doi:10.3389/fphar.2021.748956)
Supplement: Supplementary file 1 [file DataSheet1.DOCX]

Supplementary Material

**Supplementary Figure 1.** Characterization of retinal progenitor cells (RPCs)

**(A)** Undifferentiated RPCs are stained with positive markers, including PAX6, SOX2 and Nestin. RPCs are stained negative for GFAP. **(B)** Flow cytometry profiles of RPCs for subpopulations expressing PAX6, SOX2, Nestin, and GFAP, respectively.


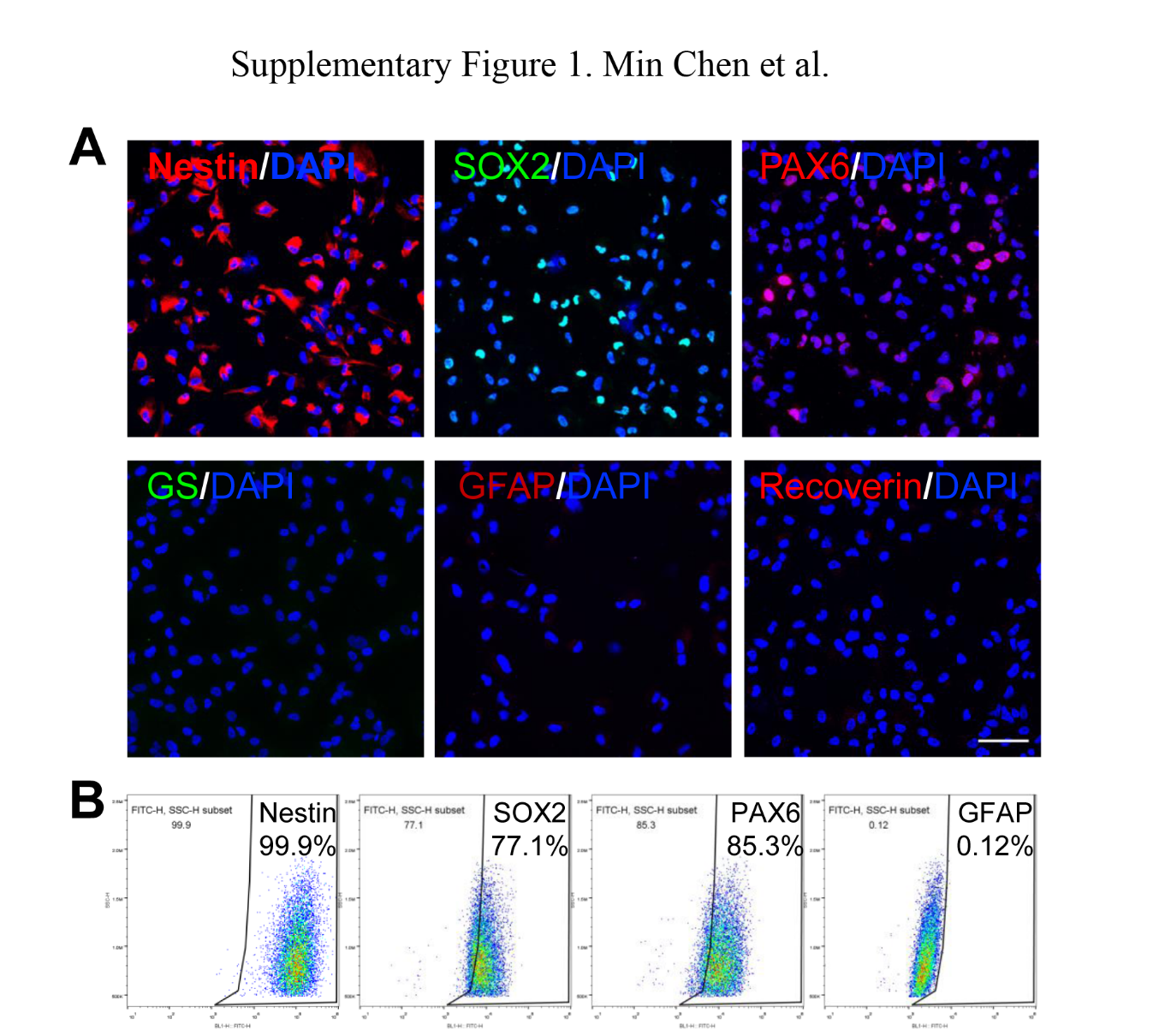


**Supplementary Figure 2.** Characterization of hRPC derived small extracellular vesicles.

(A) Identification of the main morphological characteristics of hRPC-sEVs by transmission electron microscopy (left) and enlarged view (right). Scale bar: 100 nm. **(B)** Western blot showing expression of exosomal markers: CD9, CD63 and CD81 in hRPC-sEVs. **(C)** Dynamic light scattering (DLS) and Nanoparticle tracking analysis (NTA) of sEV.


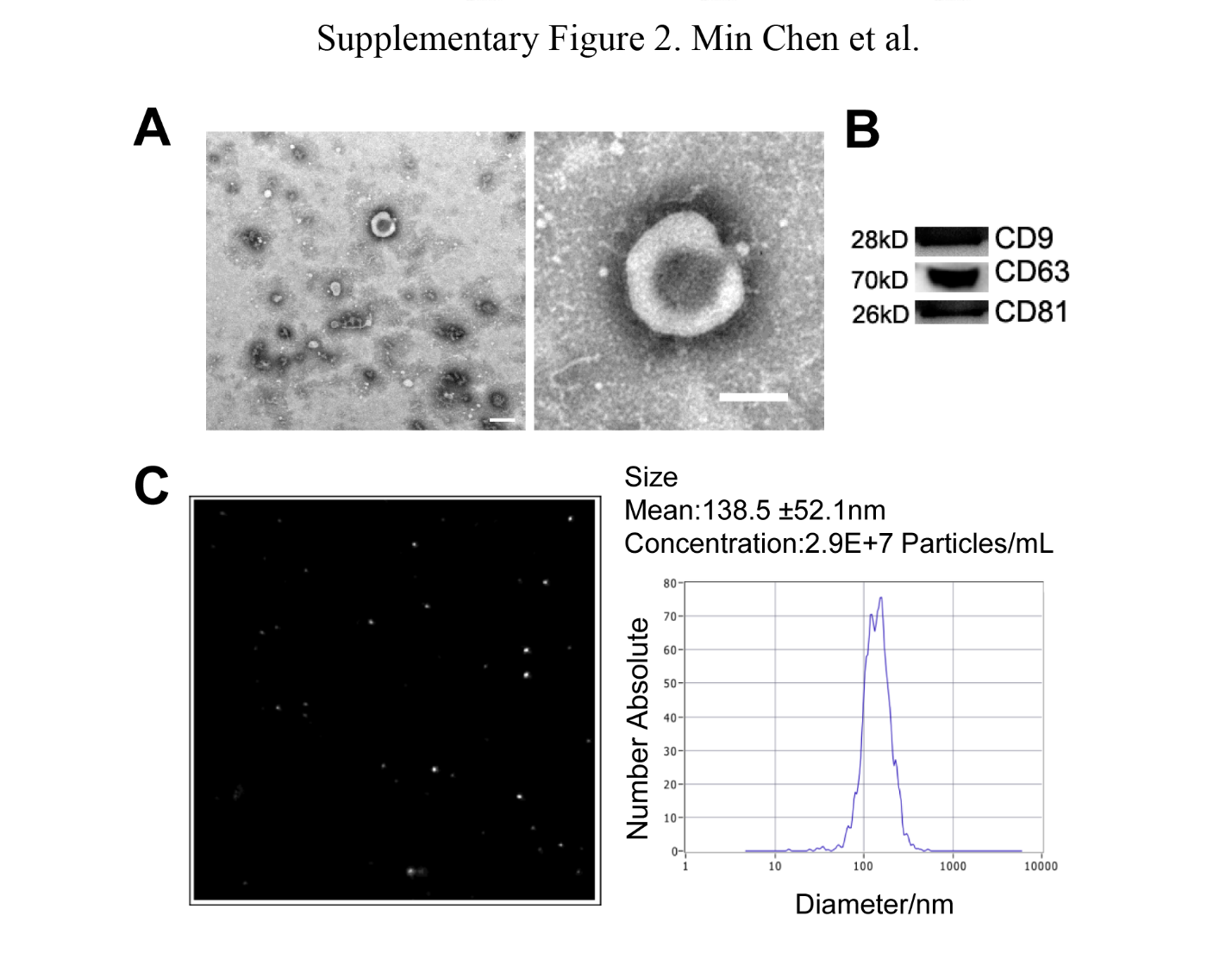


**Supplementary table 1.** Primer sequences for RT-qPCR

| primers | species | Forward | Reverse |
| --- | --- | --- | --- |
| *β-actin* | Rat | AGCCATGTACGTAGCCATCC | CTCTCAGCTGTGGTGGTGAA |
| *Bax* | Rat | TCATGAAGACAGGGGCCTTT | CTGCAGCTCCATGTTGTTGT |
| *Bcl2* | Rat | CTTCAGGGATGGGGTGAACT | ATCAAACAGAGGTCGCATGC |
| *Caspase3* | Rat | GAATCCACGAGCAGAGTCAA | CAACAAGCCAACCAAGTTCA |
| *GAPDH* | mouse | GGGTGTGAACCACGAGAAATATG | GCAGTGATGGCATGGACTGT |
| *caspase3* | mouse | GGGCCTGTTGAACTGAAAAA | CCGTCCTTTGAATTTCTCCA |
| *Bcl2* | mouse | CTGGCATCTTCTCCTTCCAG | GACGGTAGCGACGAGAGAAG |
| *Bax* | mouse | TGCAGAGGATGATTGCTGAC | GATCAGCTCGGGCACTTTAG |
| *IL-10* | Rat | CCTGCTCTTACTGGCTGGAG | TGTCCAGCTGGTCCTTCTTT |
| *IL-4* | Rat | TCCTTACGGCAACAAGGAAC | GTGAGTTCAGACCGCTGACA |
| *TGF-β* | Rat | ATACGCCTGAGTGGCTGTCT | TGGGACTGATCCCATTGATT |
| *IL-1β* | mouse | GGCAACTGTTCCTGAACTCAACTG | CCATTGAGGTGGAGAGCTTTCAGC |
| *IL-6* | mouse | TTCACAAGTCCGGACAGGAG | TGGTCTTGGTCCTTAGCCAC |
| *TNF-α* | mouse | ACGTGGAACTGGCAGAAGAG | GGTCTGGGCCATAGAACTGA |
| *GAPDH* | homo | AGGAAGATTGAGTCGCTGGA | ATACTGCGTGCGGATCTCTT |
| *IL-1β* | homo | TAGGGTAGTGCTAAGAGGA | GCGAATGACAGAGGGTTT |
| *IL-6* | homo | GAGAGTAGTGAGGAACAAGCCA | TCTTTGAGCCTGTCTTCCCC |
| *TNF-α* | homo | CCATCTATCTGGGAGGGGTCT | TGGGAAGGTTGGATGTTCGT |
| *β-actin* | homo | ACTCTTCCAGCCTTCCTTC | ATCTCCTTCTGCATCCTGTC |

**Supplementary table 2.** The TPM of the top 20 miRNAs in RPC-Exos

| **miRNA** | **RPC-Exos-1** | **RPC-Exos-2** | **RPC-Exos-3** |
| --- | --- | --- | --- |
| hsa-miR-21-5p | 243878.4156 | 345438.7726 | 345069.1426 |
| hsa-let-7i-5p | 100391.5959 | 120974.4355 | 104648.2457 |
| hsa-miR-100-5p | 68965.56569 | 39673.82959 | 41971.93338 |
| hsa-miR-148a-3p | 54608.45325 | 11922.88125 | 12117.23266 |
| hsa-miR-151a-3p | 42177.70615 | 20066.52755 | 21546.58229 |
| hsa-miR-3529-3p | 38993.79376 | 15705.65413 | 19100.60874 |
| hsa-miR-7-5p | 38993.79376 | 15705.65413 | 19100.60874 |
| hsa-miR-26a-5p | 30636.375 | 41245.38563 | 38807.91108 |
| hsa-miR-30a-5p | 22987.11682 | 9254.561652 | 12832.72152 |
| hsa-let-7g-5p | 21594.68205 | 12409.70275 | 14222.74103 |
| hsa-miR-155-5p | 16353.73182 | 20900.06786 | 24932.03496 |
| hsa-let-7f-5p | 13671.43317 | 13188.75867 | 11548.93739 |
| hsa-miR-221-3p | 13325.78337 | 14132.68292 | 19959.45136 |
| hsa-miR-381-3p | 10470.65981 | 5458.344566 | 6139.892792 |
| hsa-miR-92a-3p | 10379.32958 | 9306.215677 | 8868.734033 |
| hsa-miR-9-5p | 10236.01137 | 17715.20801 | 16922.14355 |
| hsa-miR-30d-5p | 9777.95513 | 14321.60928 | 15085.42348 |
| hsa-miR-27b-3p | 8516.19285 | 3056.786171 | 3517.286928 |
| hsa-miR-181a-5p | 8475.445516 | 11580.40798 | 14382.73406 |
| hsa-miR-99a-5p | 7268.481375 | 5244.65257 | 5240.091951 |
